# Arsenic levels in tube-wells water, food, residents’ urine and the prevalence of skin lesions in Yatenga province, Burkina Faso

**DOI:** 10.2478/v10102-012-0007-4

**Published:** 2012-03

**Authors:** Issa T. Somé, Abdoul K. Sakira, Moustapha Ouédraogo, Theodore Z. Ouédraogo, Adama Traoré, Blaise Sondo, Pierre I. Guissou

**Affiliations:** 1 Laboratoire de Chimie Analytique et de Toxicologie, UFR/ Sciences de la Santé, Université de Ouagadougou, Burkina Faso; 2 Laboratoire de Pharmacologie et de Toxicologie, UFR/ Sciences de la Santé, Université de Ouagadougou, Burkina Faso; 3 Département de Santé publique, UFR/ Sciences de la Santé, Université de Ouagadougou, Burkina Faso; 4 Service de Dermatologie et de Vénéréologie, Centre Hospitalier Universitaire Yalgado Ouédraogo de Ouagadougou, Burkina Faso

**Keywords:** arsenic, poisoning, water, skin lesion, Burkina Faso

## Abstract

The aim of the present study was to evaluate the levels of arsenic in tube-well water, food and residents’ urines samples in Yatenga province, Burkina Faso. The prevalence of skin lesions was evaluated as well. The study was cross-sectional in design. It was conducted during April 2009. Permanent residents of 20 villages were included in the study. Water samples were collected from 31 tube-wells located in the selected villages. Tomatoes, cabbages, and potatoes produced in the selected village were randomly sampled. Arsenic content in water, food, and residents’ urine was determined by atomic absorption spectrophotometry using hydride generation method. Finally, 240 people were examined by a medical doctor for skin lesions. Arsenic concentrations from the tube-well water ranged from 1 to 124 μg/l. Arsenic concentrations of more than one-half (52%) of the water samples exceeded the WHO guideline value (10 μg/l). No trace of arsenic was found in the samples of tomatoes, cabbages, and potatoes. Variation in arsenic concentrations in the urines was correlated to arsenic concentrations in tube-well water. Clinical examinations revealed that melanosis and keratosis were respectively identified in 29.26% and 46.34% of the population. Both conditions were observed in 24.39% of the population. The frequency of skin lesions was positively associated with the arsenic concentration in tube-well water. A great majority (89.53%) of those who had skin lesions were at least 18 years old. In conclusion, chronic arsenic poisoning remains a major public health problem in the province of Yatenga (Burkina Faso).

## Introduction

While the world population increases beyond 6 billions, one of the most fundamental resources for human survival, clean water, is decreasing. The rising demands for sanitary water often cannot be met by surface water supplies. Besides, surface water sources are often contaminated with microorganisms, causing a significant burden of disease and mortality (Smith *et al.*, 2000). Groundwater as the source of drinking water is usually preferred (Schmoll *et al.*, 2006). In Burkina Faso, especially in its northern part, dug wells are already widely used by many villagers and are the traditional sources of drinking water. They are usually less than 25 m deep. As the overburden is usually clay, groundwater yields from these dug wells are typically small. These traditional dug wells are potentially problematic because they are vulnerable to microbes and to drying out in the dry season (Smedley *et al.*, 2007). Consequently, within the last two decades, new borehole-drilling programs have been undertaken by administrative authorities. Many of the villages now have boreholes with hand pumps which extract groundwater for domestic purposes. These tube-wells (boreholes) are typically 50–120 m deep and most of them intercept groundwater from fractures in the basement (Smedley *et al.*, 2007). In this area of Burkina Faso, the problem of arsenic-contaminated water has only recently come to light, like in India and Bangladesh (Bagla & Kaiser, 1996; Nordstrom, 2002; Smedley & Kinniburgh, 2002).

The presence of arsenic in natural water is related to the process of leaching from the arsenic-containing rocks (Nordstrom, 2002). Arsenic is a primary constituent of certain ores and occurs as a trace impurity in others (Lorenzen *et al.*, 1995).

Adverse health effects of arsenic depend strongly on the dose and duration of exposure (Mukherjee *et al.*, 2006). More than 700,000 people in the South and East Asian region have been affected by arsenic-related diseases, especially skin and internal (lung, bladder, kidney) cancers (Rahman *et al.*, 2009; Schmoll *et al.*, 2006). Although weakness, anemia, burning sensation of eyes, solid swelling of legs, liver fibrosis, chronic lung disease, gangrene of toes, neuropathy, and skin cancer are some of the other manifestations (Guha Mazumder, 2003; Singh *et al.*, 2011), specific dermatological effects are signs of chronic exposure to arsenic. Dermatological features are melanosis (hyperpigmentation) and keratosis (rough, dry, papular skin lesions) (Mukherjee *et al.*, 2006).

The present study aimed at evaluation the levels of arsenic in tube-well water, food and residents’ urines samples in Yatenga province, Burkina Faso. The prevalence of skin lesions was evaluated as well.

## Material and methods

### Study area

Our study was conducted in the province of Yatenga. This province with a population of 444 563 is located in the Northern part of Burkina Faso. Its area is 7103 km^2^ and includes 406 villages. People directly using groundwater from tube-wells were included in the study. Twenty (20) villages were randomly selected among 116 exposed villages (according to a previous unpublished study).

### Sampling

The study was cross-sectional in design. In each of the 20 villages, 5 households were randomly selected, and then all members of these households who consented to participate in the study were included. Informed consent was obtained from all the participants in an ethical manner.

The water samples were collected from 31 tube-wells located in the selected villages during April (dry season) 2009. Water was pumped from the tube-wells for 10 to 15 min before sample collection, in order to flush out all retained water in the pipes. All the water samples were collected in polypropylene bottles and were immediately acidified with a concentrated solution of hydrogen chloride (37% v/v). Previously, polypropylene bottles were soaked in a hydrogen chloride bath and then washed with Milli-Q water. Tomatoes, cabbages, and potatoes produced in some selected villages were randomly sampled. They were dried by heating at 80 °C. Urine samples were obtained from selected people (240) and treated in the same way as water samples. The samples were stored in cold boxes in the field, then kept in a deep freezer (–20 °C) until chemical analysis.

### Sample treatment and analysis

All the samples were digested with a mixture of hydrogen chloride and nitric acid (3:1) in a microwave digester. Arsenic content in water, food, and urine was then determined by atomic absorption spectrophotometry using hydride generation method (Varian 240 AA Zeeman, USA). Milli-Q water acidified with hydrogen chloride was used as control. A commercial standard solution of arsenic was used for linearity.

### Clinical examination

Finally, 240 people were included in the study. They were examined by a medical doctor for skin lesions: melanosis (hyperpigmentation) and keratosis (rough, dry, papular skin lesions). In addition, structured questionnaires were used to collect information on socio-demographic characteristics of the series.

### Statistical analysis

Statistical analysis was performed using SPSS (version 12.0, SPSS Inc., Chicago, IL, USA) for Windows. Chi-square test was used to examine the correlation between arsenic concentration in tube-well water and frequency of skin lesions. A probability value of *p<*0.05 was considered as statistically significant in this study.

## Results

### Socio-demographic characteristics of the included residents

Table 1 shows some socio-demographic characteristics of the people included in our study. The most frequently represented were farmers (71.7%), followed by those who did not work, i.e young children and disabled people (27.9%). Tube-well water was drunk by 86.6% of the people, whereas 25.5% used both tube-wells and dug-well water.

**Table 1:** Distribution of people (n=240) from Yatenga province (Burkina Faso) included in the survey on arsenic intoxication, based on age and sex.

Age (years)	Male (%)	Female (%)	Total (%)
0–6	13.3	8.3	21.6
6–18	18.3	8	26.3
≥18	28	27.1	52.1
Total (%)	56.6	43.4	100

### Arsenic concentration in tube-well water, food and human urine samples

Arsenic concentrations in the tube-well water ranged from 1 to 124 μg/l (median, 18 μg/l) (Table 2). No trace of arsenic was found in the samples of tomatoes, cabbages, and potatoes. The arsenic concentration in the urine of 234 (97%) residents was less than the guideline value (40 μg/l) (Table 3). Variation in arsenic concentrations in the urine was correlated to arsenic concentrations in tube-well water (Table 4).

**Table 2:** Arsenic concentrations (μg/l) in tube-well water from Yatenga province, Burkina Faso (n = 31).

Range of arsenic concentrations (μg/l) in tube-wells (n = 31)	Frequency (%)	Cumulative frequency (%)
0–10	48.4	48.4
10–50	35.5	83.9
50–100	9.7	93.6
More than 100	6.4	100

**Table 3:** Distribution (%) of arsenic levels in urine samples of residents (n=240) versus arsenic concentrations in tube-well water in Yatenga province, Burkina Faso.

	Arsenic levels (μg/l) in urine samples			
Arsenic concentrations in tube-well water	< 1	1–10	10– 40	≥ 40
< 10 μg/l	47.37	50.00	2.63	0.00
≥ 10 μg/l	22.73	54.62	19.23	3.38

**Table 4:** Distribution of skin lesions in the study population (n=240) versus arsenic levels in tube-wells water in Yatenga province, Burkina Faso.

Arsenic concentration in tube-well water	Frequency of melanosis (%)	Frequency of keratosis (%)	Frequency of melanosis and keratosis (%)
< 10 μg/l	25.40	33.33	20.63
≥ 10 μg/l	42.10*	89.47*	36.84*

* Difference was significant (*p*<0.001) using Chi-square test

### Skin lesions

The clinical examinations revealed that melanosis and keratosis were respectively found among 29.26% and 46.34% of the population. Both signs were simultaneously observed among 24.39% of the population. Keratosis was frequently located in the foot palm (83.72%), followed by the hand palm (55.81%), and in both foot and hand palm (46.1%). Melanosis was frequently observed on the limbs, then on the chest or back.

The frequency of skin lesions (melanosis or keratosis) was positively associated with arsenic concentration in tube-well water used by the population (Table 4). The prevalence of skin lesions increased with age. A great majority (89.53%) of those who had skin lesions was at least 18 years old; 9.30% were between 6 and 18 years old.

## Discussion

Arsenic concentrations of more than one-half (52%) of the water samples exceeded the World Health Organization (WHO) guideline value (10 μg/l) (WHO, 2004). A previous study (not published) carried out in the northern region of Burkina Faso in 2004 showed that only 13% of the boreholes water samples had abnormal arsenic concentrations (>10 μg/l). In the present study, high arsenic concentrations among most of the tube-well water samples might reflect the oxidation/weathering of arsenopyrite. In addition, the analytical procedure in the previous study used a field testing kit whose accuracy is questionable (Masud, 2000). The variation of arsenic levels in tube-well water might also be related to pH and Eh variation in aquifer; higher concentrations of arsenic were detected in groundwater with pH>6.7 and slightly reducing conditions (Eh 200–300 mV) (Smedley, 1996). Therefore it will be necessary to check also these parameters in tube-well water.

In Bangladesh, 17 out of 52 districts had a maximum level of arsenic in groundwater exceeding 1 mg/l. The concentration detected in some areas of this country was 14 mg/l (The New Nation, 1997). In Gia Lam District and Thanh Tri District, suburban areas of Hanoi, Vietnam, in September 2001, concentrations of arsenic in the groundwater ranged from <0.10 to 330 μg/l, with about 40% of samples exceeding by WHO drinking water guideline value (Agusa *et al.*, 2006).

The absence or undetectable level of arsenic in food samples is understandable because the vegetables were watered with water from dug well or rivers. In high-arsenic areas, dug well water may contain less arsenic than tube-wells water (Smedley, 1996, 2003). Human exposure of arsenic through the consumption of contaminated drinking water in these areas may cause serious health problems.

Presence of arsenic in urine or body tissue samples without symptoms is the first stage of chronic arsenic poisoning (Choong *et al.*, 2007). Concentrations of arsenic in human urine increased significantly with those in water, implicating that the source of arsenic in humans might be drinking water. A similar trend was also observed in Bangladesh, India, and in Ghana (Asante *et al.*, 2007). In addition, more than 3% of the collected urine samples showed a heavy concentration of arsenic. The normal amount of arsenic in urine ranges from 0.005 to 0.040 mg/day (assuming the total discharge of urine in one day is 1.5 l) (Farmer & Johnson, 1990).

The prevalence of skin lesions increased with the concentration of arsenic in drinking water from tube-wells. However, this prevalence was lower than the prevalence of abnormal concentrations of arsenic in residents’ urine and in water samples though it is well-known that chronic arsenic exposure causes skin lesions (Abernathy *et al.*, 1999; Tsunetoshi, 2000). Previous studies reported that there was no symptom of arsenic poisoning in residents from the suburb of Hanoi, Vietnam, in spite of the high concentrations of arsenic in groundwater (Agusa *et al.*, 2006; Berg *et al.*, 2001). Indeed, nutritional status and genetic polymorphism may also influence the expression of arsenic toxicity. Low intake of calcium, animal protein, folate, and fiber may increase susceptibility to arsenic-caused skin lesions (Mitra *et al.*, 2004). Furthermore, a large variation in the susceptibility to arsenic toxicity between individuals and ethnic groups may be associated with genetic factors in arsenic metabolism (Vahter, 2002).

Skin lesions were rare among children who were under 6 years old. This is not surprising because melanosis and keratosis of the hands or feet usually appear after 5–15 years of arsenic exposure (Agusa *et al.*, 2009; Tseng, 1977).

## Conclusion

Our study revealed that the level of arsenic in the water of most tube-wells was over the World Health Organization (WHO) guideline value, suggesting the need for research on contamination of multiple elements in tube-well water and their mixture toxicity. However, vegetables were not contaminated by arsenic. Arsenic concentrations in the residents’ urine were correlated with arsenic concentrations in tube-well water. Melanosis and keratosis were observed in most residents whose drinking water was most contaminated by arsenic.

As studies showed that chronic exposure to arsenic may also cause reproductive, neurological, cardiovascular, respiratory, hepatic, hematological, and diabetic problems in humans, further studies are needed to evaluate the potential health effects of arsenic from tube-well water in this area (Yatenga province, Burkina Faso).

## Acknowledgement

The authors are grateful to the Embassy of Denmark in Burkina Faso and PADSEA (Programme d'Appui au Développement du Secteur Eau et Assainissement) for their financial and technical assistance.
